# The impact of social loafing on college students’ classroom silence: a moderated mediation model

**DOI:** 10.3389/fpsyg.2025.1682073

**Published:** 2025-11-19

**Authors:** Shanshan Li, Hongyi Zhou, Yuannan Zheng

**Affiliations:** 1 School of Economics and Management, Anhui University of Science and Technology, Huainan, China; 2 School of Safety Science and Engineering, Anhui University of Science and Technology, Huainan, China

**Keywords:** social loafing, classroom silence, learning motivation, instructional factors, a moderated mediation model

## Abstract

The phenomenon of classroom silence significantly impacts the enhancement of instructional quality. Investigating its causes and pathways constitutes the core breakthrough in addressing this issue. Adopting social loafing as the independent variable, this study constructs a theoretical model mediated by learning motivation and moderated by instructional factors. Through questionnaire surveys and structural equation modeling analysis involving 1,402 college students, we explore the influential pathways of classroom silence. Key findings include: (1) Social loafing positively predicts classroom silence (*β* = 0.42), with learning motivation serving as a partial mediator, accounting for 11.55% of the mediating effect; (2) Instructional factors demonstrate differentiated moderating mechanisms: content and methodology moderate the mediator model through dual-path regulation—mitigating social loafing’s negative impact on learning motivation (moderating effects *β* = −0.02 ~ −0.03) while reinforcing learning motivation’s inhibitory effect on silence (*β* = −0.02). Teaching style uniquely regulates the first pathway, whereas instructor characteristics systematically regulate the entire mediation process; (3) Although quality instructional elements reduce silent behaviors through mediation pathways, they paradoxically amplify the direct effects of social loafing among individuals, highlighting group differentiation phenomena. This study provides practical guidance for higher education management and theoretical references for mitigating college students’ silent classroom behaviors.

## 1 Introduction

In recent years, a key focus of higher education reform in China has been the promotion of heuristic, interactive, and inquiry-based teaching methodologies. This shift moves away from teacher-dominated instruction toward guiding students to actively participate, engage in interaction, and engage in continuous critical thinking, thereby cultivating their capacity for independent inquiry and innovation (People's Education, 2019). The active participation of university students in the classroom constitutes a crucial metric for evaluating teaching effectiveness (Ning, 2016). Interactions between instructors and students during class can facilitate psychological and cognitive processing, stimulate ongoing reflection, and influence the construction of knowledge frameworks, ultimately enriching students’ knowledge systems and fostering the sound development of their cognitive abilities (Jiang et al., 2022).

However, a prevalent phenomenon persists within current university classrooms: instructor-dominated monologue (“teacher-centered cramming”) coupled with student disengagement, manifested as silence and a reluctance to participate in classroom interaction. This occurrence of classroom silence significantly diminishes teaching quality. Classroom silence essentially refers to students’ unwillingness or perceived inability to interact with the instructor, their hesitation or refusal to express their views, and their adoption of a silent demeanor during class, a behavior which also embodies significant educational implications (Li and Tao, 2022). On one hand, student silence in class impedes the development of independent thinking and verbal expression skills. On the other hand, pervasive classroom silence hinders instructors’ ability to gauge student comprehension, discourages educators from implementing pedagogical reforms, leads to inefficient utilization of educational resources, and consequently severely compromises teaching quality.

To mitigate the occurrence of classroom silence among university students and continuously enhance the openness and effectiveness of university teaching, extensive research in recent years has focused on exploring its underlying causes. The reasons behind student silence in university classrooms are complex. Current research primarily examines influencing factors at multiple levels, including the student, instructor, and external environment; however, the interrelationships among these factors remain inadequately understood. Within the context of the “Double First-Class” Initiative, a thorough investigation into the correlational relationships between the causes of university classroom silence and corresponding countermeasures holds significant practical importance for effectively addressing this issue and establishing high-quality teaching practices in higher education institutions.

Therefore, this study employs social loafing as a theoretical lens to probe its relationship with classroom silence. Furthermore, it positions learning motivation as a mediating variable and instructional factors as moderating variables, conducting an in-depth analysis of their interrelationships. This approach aims to propose targeted solutions, offering valuable insights for breaking the pattern of classroom silence among university students within the higher education teaching process.

## 2 Literature review

### 2.1 Social loafing

Social loafing, initially proposed by German psychologist Max Ringelmann in 1913, refers to the phenomenon where individuals exert less effort and demonstrate reduced motivation when working collectively compared to performing tasks independently. This manifests as a decrease in individual contribution as group size increases. Contemporary researchers broadly categorize the causes of social loafing into three dimensions: individual factors, group factors, and multilevel contextual factors. Mulvey and Klein’s (1998) study found that individuals tend to reduce effort when observing peers engaging in free-riding behavior. Li and Yan (2011) empirically demonstrated through creative task experiments that setting challenging goals and evaluating individual contributions effectively mitigate social loafing. Ma and Wei (2005) identified subjective willingness to collaborate as a primary individual-level determinant of social loafing. Ma’s (2006) research revealed that individuals frequently project their assumptions onto group members, reducing personal effort based on perceived peer underperformance. Lin and Huang (2009) discovered organizational justice and organizational trust as critical inhibitors of social loafing in knowledge-sharing contexts, noting that enhanced interactional justice significantly reduces knowledge withholding behaviors. Liu et al. (2024) demonstrated that among employees facing high job demands or possessing strong moral identity, morning social loafing paradoxically improves afternoon performance through recovery mechanisms and guilt-induced compensation. Chen and Orly’s (2023) study established a positive correlation between individual and group organizational citizenship behaviors (OCB), while revealing an inverse relationship between group OCB and social loafing.

### 2.2 Learning motivation

Learning motivation is widely regarded as an intrinsic psychological driver of classroom silence, the absence or diminishment of which directly leads to students’ reluctance or hesitation to participate in classroom interactions. Research by Chen et al. (2025) revealed that learning motivation partially mediates the relationship between teacher-student relationships and postgraduate students’ satisfaction with their supervisors, with supervisory mentoring styles moderating the connection between learning motivation and teacher-student dynamics. Lee’s (2025) study demonstrated that a Structured News Case Method significantly enhanced students’ learning motivation, critical thinking, problem-solving abilities, and financial news awareness, though its impact on theoretical understanding showed no significant difference compared to traditional lectures. Huang et al.’s (2025) research on university students indicated that intrinsic motivation positively correlated with GPA both directly and indirectly through increased use of effective learning strategies and reduced perceived stress. Extrinsic motivation influenced GPA indirectly via strategic learning behaviors, while amotivation exhibited the strongest negative correlation with GPA, operating through diminished strategy use and heightened stress. Ye et al.’s (2025) investigation of Chinese vocational college students found that students with stronger self-leadership capabilities were more likely to exhibit proactive learning motivation and experience higher levels of motivational engagement during learning. Eticha et al.’s (2025) study showed that a designed problem-solving approach incorporating metacognitive scaffolds significantly improved students’ learning motivation and academic achievement, suggesting the need for further research on its integration into secondary school biology instruction. Wood and Tribe (2016) innovatively proposed that classroom silence is essentially an active behavioral choice made by students based on subjective judgment. Zhang et al. (2019) proposed a dual-system theoretical model of classroom interaction, attributing silence to the dynamic interplay between an obstructive system (comprising ability deficits, group anxiety, and apathy) and a driving system (comprising achievement motivation, subject interest, and learning perseverance).

### 2.3 Classroom silence

Classroom silence emerges as a multifaceted phenomenon arising from structural-cultural factors, intertwined with individual psychological and behavioral elements, and further compounded by instructional design and environmental triggering mechanisms. Based on behavioral psychology, Zhang (2019) classified classroom silence into “Inability-Induced Silence” (stemming from insufficient knowledge reserves) and “Resistance-Induced Silence” (triggered by psychological defense mechanisms). Cui and Zhu (2022) further argued for the moderating effects of student personality trait differences and cognitive ability levels on the quality of teacher-student interaction. The influence of cultural and environmental mechanisms has also garnered scholarly attention. From a cultural sociology perspective, Lv (2020) explained that Confucian cultural traditions emphasizing teacher authority shape student behaviors that avoid challenging this authority in the classroom. Zhang and Shi (2020) found that stable teaching belief systems formed through traditional cultural accumulation exert a latent regulatory effect on students’ classroom expression behaviors. Shi (2020) pointed out that the “teacher-dominated discourse power” rule system formed in traditional classroom settings reinforces students’ habitual silence. Dong and Zhao (2023) analyzed unequal class culture, indifferent relational culture, and unreasonable competitive culture as significant causes of classroom silence.

### 2.4 Instructional factors

Instructional factors are actionable and modifiable external variables; optimizing which can directly or indirectly reduce the occurrence rates of classroom silence. Wang et al. (2023) found that teachers’ personal characteristics exerted a significant influence on student silence, confirming the instructor’s role during lectures as a primary factor affecting this phenomenon. Yang (2024) identified several triggers for classroom silence, including: teachers’ lack of proper understanding of silence, teaching content misaligned with students’ developmental levels and needs, inappropriate question difficulty, topics mismatched with student interests, and teaching materials failing to meet practical student requirements. Jia et al. (2021) demonstrated that both teaching methods and student literacy are significant factors influencing the degree of passive classroom silence. Wendt and Courdu (2018) revealed that the educational models employed by university teachers not only significantly impact students’ classroom participation enthusiasm but also shape their cognitive structures through long-term effects. Osman et al. (2022) confirmed that effective motivational strategies implemented by teachers during instruction can significantly increase student interaction frequency. Guo et al. (2025) through machine learning optimization and validation, substantiated that teaching content, delivery methods, and assessment management all influence the occurrence of silent classroom behavior.

Within the tradition of higher education research, “teaching behaviors” are systematically categorized into two distinct tiers: situational strategies and cross-situational dispositions, wherein “instructional style” refers to teaching techniques—adjustable within specific instructional units—that activate students’ cognitive engagement (e.g., case introductions, problem-driven activities, immediate feedback, peer assessment), exhibiting state-like variability across topics and sessions; conversely, “teaching style” reflects instructors’ stable personal characteristics and emotional expressions (e.g., enthusiasm, humor, rapport, verbal expressiveness) that remain consistent across courses and semesters, representing a trait-like dimension. This state–trait distinction is theoretically critical in teacher effectiveness models, as these factors operate through divergent psychological mechanisms: situational strategies primarily enhance cognitive involvement, whereas affective dispositions strengthen learning motivation by fulfilling belongingness and identity needs (Wendt and Courdu, 2018). Merging them into a single factor would obscure their differential impact pathways and mask their unique moderating effects on the “social loafing → learning motivation → classroom silence” chain. Consequently, retaining both as independent moderators aligns with international theoretical conventions and culturally grounded construct boundaries in the Chinese context.

### 2.5 Interrelationship

The causes of classroom silence among university students are characterized by multifaceted complexity, where learning motivation, social loafing, and instructional factors collectively exert influence, manifesting through classroom climate, student agency, and cultural environment. Peng et al. (2023) discovered a significant positive correlation between the cultural distance experienced by migrant students and their perceived classroom learning gains, noting that cultural distance acts as a protective mechanism increasing their silence. Li and Ye (2022) identified student self-perception and core competencies as major factors influencing classroom silence. Frambach et al. (2014) constructed an influence model encompassing multi-dimensional elements such as cultural traditions, organizational relationships, and class characteristics. Muuro et al.’s (2014) confirmed that physical environmental elements, including teaching equipment configuration and peer participation levels, have significant moderating effects on classroom interaction frequency. Gao (2020) from an educational ecology perspective, differentiated the dual mechanisms of explicit environmental elements (e.g., classroom spatial layout, class size) and implicit atmosphere elements (e.g., class cohesion, attention focus). He (2020) demonstrated that traditional classroom atmospheres positively influence silence. Lv (2018) also posited that authentic educational contexts are influencing factors for student silence. Liu et al. (2021) found that excessively large class sizes hinder teachers’ individual attention to students, leading to the neglect of individual differences, reduced teacher-student interaction, and consequently, classroom silence. Additionally, Zhao and Liu (2024) identified technology application and classroom format as significant contributors to silence. Chen (2023) highlighted classroom atmosphere and interaction methods as crucial factors influencing student silence.

Current research on social loafing, learning motivation, and classroom silence primarily focuses on single dimensions or fragmented aspects, lacking investigation into the interrelationships among the various influencing factors of college students’ classroom silence, especially the interactive pathways between multiple factors. Secondly, the dominance of cross-sectional designs limits causal inference, with insufficient attention paid to the “state–trait” attributes of silence and its temporal evolution mechanisms. Therefore, this study takes social loafing as the starting point, delving deeply into its relationship with classroom silence. It positions learning motivation as a mediating variable and teaching factors as moderating variables to conduct an in-depth analysis of their interrelationships. The aim is to propose targeted solutions, providing insights for breaking college students’ classroom silence within higher education settings.

## 3 Research design

This study strictly complied with local legislation and institutional requirements. All participants provided written informed consent indicating their voluntary participation. Participants were informed of the research purpose, voluntary nature, anonymity guarantee, and data handling procedures through a written disclosure statement at the beginning of the questionnaire, and were advised that they could withdraw at any time without penalty. All procedures were conducted in accordance with local data protection regulations. No personally identifiable information was collected. Any quoted statements or aggregated statistics presented in this paper are based on anonymized coded data. The full questionnaire (including target scales) required approximately 8–10 min to complete.

### 3.1 Research scale

Social loafing: measured using the scale developed by George (1992), comprising 10 items. As George’s study targeted department store employees while this research focuses on college students, the wording was slightly adapted without altering the original meaning to enhance suitability for the student population. For instance, the item “When other coworkers are working on this task’ was revised” to “When other members are working on this task.” Responses were recorded on a Likert 5-point scale. The overall Cronbach’s *α* for this scale was 0.91.

Classroom silence: assessed using the Classroom Silence Behavior Questionnaire developed by Liu (2020), consisting of 8 items. Responses were recorded on a Likert 5-point scale. The overall Cronbach’s *α* for this scale was 0.91.

Learning motivation: adapted from the Academic Motivation Scale revised by Chi and Xin (2006). This 10-item scale includes two dimensions, extrinsic motivation (5 items, Cronbach’s *α* = 0.79), e.g., “Higher education better prepares me for my career”; intrinsic motivation (5 items, Cronbach’s α = 0.87), e.g., “Higher education allows me to experience joy and fulfillment through knowledge growth in my field of passion.” Responses were recorded on a Likert 5-point scale. The overall Cronbach’s α was 0.87.

Instructional factors: adapted from the Teaching Style Scale developed by He (2005). The 13-item scale comprises four dimensions. Instructional content (3 items, α = 0.84), e.g., “The instructor prioritizes sharing practical skills and operational techniques”; instructional methods (3 items, α = 0.84), e.g., “Teachers focus more on sharing practical skills and teaching practical methods or techniques in the classroom”; instructional style (3 items, α = 0.82), e.g., “The instructor encourages creative problem-solving through novel approaches”; Teaching style (4 items, α = 0.88), e.g., “The instructor delivers lectures with enthusiasm, using dynamic language to foster a positive classroom atmosphere.” Responses were recorded on a Likert 5-point scale. The overall Cronbach’s α was 0.95.

### 3.2 Research sample

#### 3.2.1 Pre-survey

Prior to the formal research, a pre-survey was conducted to assess the reliability and validity of the questionnaire. Questionnaires were distributed using a combination of online and offline modes. The pre-survey targeted full-time undergraduate students, yielding an initial collection of 300 responses. Following screening and data cleaning, 28 invalid questionnaires exhibiting repeated responses or significant omissions were removed to ensure data analysis reliability. Consequently, 272 valid questionnaires were retained, representing an effective response rate of 90.67%. After screening, reliability and validity analyses were performed on the questionnaire data; the results are presented in Table 1.

**Table 1:** Reliability and validity.

	Social loafing	Classroom silence	Learning motivation	Instructional factors				
				Instructional content	Instructional methods	Instructional style	Teacher’s style	Total scale
Cronbach’s *α*	0.92	0.89	0.88	0.81	0.84	0.82	0.86	0.95
Kaiser-Meyer-Olkin (KMO) Measure	0.95	0.92	0.95	0.73	0.72	0.72	0.84	0.98

As shown in Table 1, the reliability and validity of each measurement questionnaire are ideal and can be used for formal research.

#### 3.2.2 Formal research

Considering the sample’s characteristics of wide geographic distribution and large size, a sampling survey method was adopted for the formal investigation. Data collection utilized a combination of paper-based and online questionnaires, targeting full-time undergraduate students as the research subjects. Following data collection, 1,554 questionnaires were initially retrieved. After screening and data cleaning, 152 invalid questionnaires exhibiting excessive response repetition, significant omissions, or unreasonable completion times were excluded to ensure data analysis reliability. Consequently, 1,402 valid questionnaires were obtained, representing an effective response rate of 90.21%. The detailed sample structure is presented in Table 2.

**Table 2:** Demographic variables.

Variable		Number	Percentage (%)
Gender	Male	793	56.6
	Female	609	43.4
Only child	Yes	649	46.3
	No	753	53.7
Grade	First-year of university	238	17.0
	Second-year of university	456	32.5
	Third-year of university	290	20.7
	Fourth-year of university	188	13.4
	Master	192	13.7
	Doctoral	38	2.7
Household category	Urban	634	45.2
	countryside	768	54.8
Professional category	Humanities and social sciences	644	45.9
	Science and engineering	758	54.1
Political profile	Massed	677	48.3
	Communist youth league members	524	37.4
	Provisional party members	118	8.4
	Communist party members	83	5.9

### 3.3 Theoretical model

**Figure 1:**
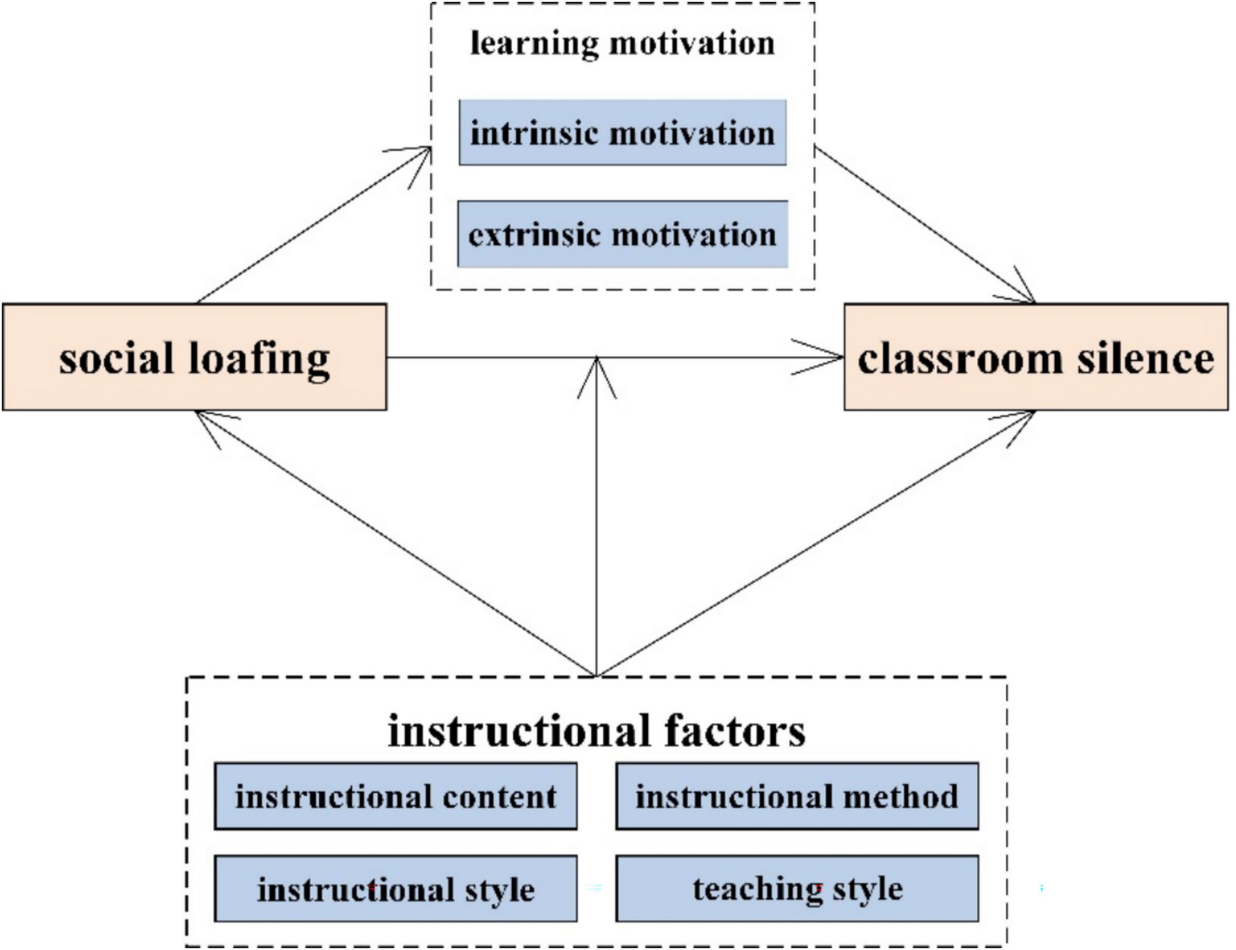
Theoretical model.

The primary objective of this study is to investigate the mechanism through which social loafing influences classroom silence behaviors among university students. Specifically, learning motivation is examined as a mediating variable, while instructional factors function as moderating variables within the theoretical framework. The conceptual model constructed for this research is presented in Figure 1.

## 4 Results and analysis

### 4.1 Confirmatory factor analysis

As shown in Table 3, the model of social loafing, learning motivation, instructional factors, and university students’ classroom silent behavior fits well overall.

**Table 3:** Confirmatory factor analysis.

χ^2^/df	RMSEA	GFI	AGFI	CFI	IFI	TLI
3.594	0.043	0.904	0.893	0.945	0.945	0.942

χ^2^/df is the chi-square-to-degrees-of-freedom ratio; RMSEA, GFI, AGFI, CFI, IFI, and TLI are all model fit indices.

### 4.2 Correlation analysis

Analysis results for social loafing, learning motivation, instructional factors, and classroom silence behaviors are presented in Table 4. The table displays descriptive statistics, Pearson correlation coefficients, composite reliability (CR), and average variance extracted (AVE). First, all variables demonstrated AVE values exceeding 0.5 and CR values above 0.8, indicating adequate convergent validity of the study’s structural model.

**Table 4:** Correlation coefficient between main variables.

variable	M ± SD	CR	AVE	1	2	3	4	5	6	7
1 social loafing	24.40 ± 8.37	0.92	0.53	1.00						
2 classroom silence	25.50 ± 7.12	0.91	0.55	0.42**	1.00					
3 learning motivation	34.24 ± 7.31	0.90	0.50	−0.35**	−0.27**	1.00				
4 instructional content	10.74 ± 2.80	0.84	0.63	−0.40**	−0.34**	0.45**	1.00			
5 instructional method	10.81 ± 2.74	0.83	0.62	−0.38**	−0.33**	0.45**	0.83**	1.00		
6 instructional style	10.64 ± 2.67	0.82	0.60	−0.39**	−0.33**	0.46**	0.82**	0.82**	1.00	
7 teaching style	14.40 ± 3.64	0.88	0.65	−0.41**	−0.34**	0.47**	0.84**	0.83**	0.84**	1.00

**Denotes *p*<0.01, *denotes *p*<0.05.

In correlation analyses, social loafing showed significant negative correlations with learning motivation, teaching content, teaching methods, teaching style, and teacher’s personal style (all *p* < 0.01), while demonstrating a significant positive correlation with classroom silence (*r* = 0.42, *p* < 0.01). Significant positive intercorrelations were observed among learning motivation, teaching content, teaching methods, teaching style, and teacher’s personal style, thereby establishing an empirical foundation for subsequent mediation and moderation model testing.

### 4.3 The mediating role of learning motivation

The total effect of social loafing on classroom silence was significant (*B* = 0.36, *β* = 0.42, *p* < 0.01). After controlling for learning motivation, the direct effect of social loafing on classroom silence remained significant (*B* = 0.32, *β* = 0.37, *p* < 0.01). Indirect effect analysis demonstrated that social loafing negatively predicted learning motivation (*B* = −0.31, *β* = −0.35, *p* < 0.01), while learning motivation negatively predicted classroom silence (*B* = −0.13, *β* = −0.14, *p* < 0.01). The indirect effect value was 0.04 (95% BootCI [0.03, 0.07]), accounting for 11.55% of the total effect. This indicates that learning motivation partially mediates the relationship between social loafing and classroom silence—that is, social loafing not only directly increases classroom silence but also indirectly aggravates silence behaviors by reducing learning motivation (standardized effect proportion: 11.55%).

### 4.4 Moderated mediation effects

After controlling for statistical variables, a moderation analysis was conducted on the four dimensions of instructional content, instructional methods, instructional style, and teaching style among the instructional factors. The results are as follows:

1. The moderating mediating effect of instructional content, as shown in Table 5.

**Table 5:** Moderated mediation modeling tests of instructional content (*N* = 1,402).

Predictor variable	Classroom silence			Learning motivation		
	*β*	*SE*	*t*	*β*	*SE*	*t*
Constant	19.79**	1.23	16.11	−2.54*	1.27	−2.00
Social loafing	0.28**	0.02	12.84	−0.17**	0.02	−7.73
Instructional content	−0.48**	0.07	−7.00	0.97**	0.068	14.57
Social loafing* instructional content	0.05**	0.01	7.13	−0.02**	0.01	−2.97
Learning motivation	−0.06*	0.02	−2.51			
Learning motivation* instructional content	−0.02*	0.01	−2.38			
*R* ^2^	0.26			0.25		
Δ*R*^2^	0.25			0.24		
*F*	*F* (11, 1,390) = 44.67, *p* = 0.000			*F* (9, 1,392) = 50.76, *p* = 0.000		

**Denotes *p* < 0.01, *denotes *p* < 0.05.

As indicated in Table 5, Model 1 (dependent variable: classroom silence) demonstrated significant overall explanatory power (*R*^2^ = 0.261, *F* (11, 1,390) = 44.67, *p* < 0.01), and Model 2 (dependent variable: learning motivation) also exhibited significant explanatory power (*R*^2^ = 0.25, *F* (9, 1,392) = 50.76, *p* < 0.01), indicating good model fit to the data. Secondly, the direct effect of social loafing on classroom silence was significant (*β* = 0.28, *t* = 12.84, *p* < 0.01), suggesting that higher levels of social loafing predict increased classroom silence behaviors. Teaching content significantly moderated the relationship between social loafing and classroom silence (*β* = 0.05, *t* = 7.13, *p* < 0.01). To clarify the interaction effect, teaching content was divided into high-level (+1SD) and low-level (-1SD) groups based on ±1 standard deviation around the mean. Simple slope analysis revealed that when teaching content quality was high (+1SD), social loafing had a stronger predictive effect on classroom silence (*β* = 0.43, 95% CI [0.37, 0.49]), whereas the effect weakened when teaching content quality was low (−1SD) (*β* = 0.14, 95% CI [0.08, 0.20]).

Moderated mediation analysis further showed that, in the first stage of the mediated pathway, teaching content negatively moderated the effect of social loafing on learning motivation (*β* = −0.02, *p* < 0.01), with higher-quality teaching content weakening the negative impact of social loafing on learning motivation; in the second stage of the mediated pathway, teaching content negatively moderated the effect of learning motivation on classroom silence (*β* = −0.02, *p* < 0.05), with higher-quality teaching content strengthening the inhibitory effect of learning motivation on classroom silence. Additionally, the moderated mediation index was statistically significant (Index = 0.005, 95% Bootstrapped CI [0.001, 0.011]), confirming that teaching content’s moderating effect on the mediated pathway holds substantive meaning.

1. The moderating mediating effect of instructional method, as shown in Table 6.

**Table 6:** Moderated mediation modeling tests of instructional method (*N* = 1,402).

predictor variable	Classroom silence			Learning motivation		
	*β*	*SE*	*t*	*β*	*SE*	*t*
Constant	19.78**	1.23	16.10	−2.84*	1.27	−2.24
Social loafing	0.29**	0.02	13.02	−0.18**	0.022	−8.00
Instructional method	−0.50**	0.07	−7.12	1.00**	0.07	14.80
Social loafing* instructional method	0.05**	0.01	6.68	−0.03**	0.01	−3.55
Learning motivation	−0.06*	0.03	−2.39			
Learning motivation* instructional method	−0.02**	0.01	−2.73			
*R* ^2^	0.26			0.25		
Δ*R*^2^	0.25			0.24		
*F*	*F* (11, 1,390) = 44.10, *p* = 0.000			*F* (9, 1,392) = 51.66, *p* = 0.000		

**Denotes *p* < 0.01, *denotes *p* < 0.05.

As indicated in Table 6, Model 1 (dependent variable: classroom silence) demonstrated significant overall explanatory power (*R*^2^ = 0.26, *F* (11, 1,390) = 44.10, *p* < 0.01), and Model 2 (dependent variable: learning motivation) also exhibited significant explanatory power (*R*^2^ = 0.25, *F* (9, 1,392) = 51.66, *p* < 0.01), indicating good model fit to the data. Secondly, the direct effect of social loafing on classroom silence was significant (*β* = 0.29, *t* = 13.02, *p* < 0.01), suggesting that higher levels of social loafing predict increased classroom silence behaviors. Teaching methods significantly moderated the relationship between social loafing and classroom silence (*β* = 0.05, *t* = 6.68, *p* < 0.01). To clarify the interaction effect, teaching methods were divided into high-level (+1SD) and low-level (−1SD) groups based on ±1 standard deviation around the mean. Simple slope analysis revealed that when teaching methods quality was high (+1SD), social loafing had a stronger predictive effect on classroom silence (*β* = 0.42, 95% CI [0.36, 0.48]), whereas the effect weakened when teaching methods quality was low (−1SD) (*β* = 0.15, 95% CI [0.10, 0.21]).

Moderated mediation analysis further showed, in the first stage of the mediated pathway, teaching methods significantly and negatively moderated the effect of social loafing on learning motivation (*β* = −0.03, *t* = −3.55, *p* < 0.01), indicating that higher-level teaching methods can mitigate the negative impact of social loafing on learning motivation; in the second stage of the mediated pathway, teaching methods further negatively moderated the effect of learning motivation on classroom silence (*β* = −0.02, *t* = −2.73, *p* < 0.05), demonstrating that high-level teaching methods strengthen the inhibitory effect of learning motivation on classroom silence. Additionally, the moderated mediation index was statistically significant (Index = 0.005, 95% CI [0.002, 0.012]).

1. The moderating mediating effect of instructional style, as shown in Table 6.

As indicated in Table 7, Model 1 (dependent variable: classroom silence) demonstrated significant overall explanatory power (*R*^2^ = 0.26, *F* (11, 1,390) = 45.34, *p* < 0.01), and Model 2 (dependent variable: learning motivation) also exhibited significant explanatory power (*R*^2^ = 0.25, *F* (9, 1,392) = 52.37, *p* < 0.01), indicating good model fit to the data. Secondly, the direct effect of social loafing on classroom silence was significant (*β* = 0.29, *t* = 13.16, *p* < 0.01), suggesting that higher levels of social loafing predict increased classroom silence behaviors. Teaching style significantly moderated the relationship between social loafing and classroom silence (*β* = 0.05, *t* = 7.69, *p* < 0.01). To clarify the interaction effect, teaching style was divided into high-level (+1SD) and low-level (−1SD) groups based on ±1 standard deviation around the mean. Simple slope analysis revealed that when teaching style quality was high (+1SD), social loafing had a stronger predictive effect on classroom silence (*β* = 0.42, 95% CI [0.38, 0.50]), whereas the effect weakened when teaching style quality was low (−1SD) (*β* = 0.15, 95% CI [0.08, 0.19]).

**Table 7:** Moderated mediation modeling tests of instructional style (*N* = 1,402).

Predictor variable	Classroom silence			Learning motivation		
	*β*	*SE*	*t*	*β*	*SE*	*t*
Constant	20.03**	1.23	16.33	−2.77*	1.27	−2.19
Social loafing	0.29**	0.02	13.16	−0.17**	0.02	−7.83
Instructional style	−0.50**	0.07	−6.96	1.03**	0.07	14.90
Social loafing * instructional style	0.06**	0.01	7.69	−0.03**	0.01	−3.58
Learning motivation	−0.06*	0.03	−2.20			
Learning motivation * instructional style	−0.02	0.01	−1.90			
*R* ^2^	0.26			0.25		
Δ*R*^2^	0.26			0.25		
*F*	*F* (11, 1,390) = 45.34, *p* = 0.000			*F* (9, 1,392) = 52.37, *p* = 0.000		

**Denotes *p* < 0.01, *denotes *p* < 0.05.

Moderated mediation analysis further showed, in the first stage of the mediated pathway, social loafing significantly and negatively predicted learning motivation (*β* = −0.17, *t* = −7.83, *p* < 0.01), while the interaction term of social loafing × teaching style was significant (*β* = −0.026, *t* = −3.58, *p* < 0.01). This indicates that teaching style buffered the negative impact of social loafing on learning motivation, meaning optimized teaching styles can mitigate the detrimental effects of social loafing on learning motivation. in the second stage of the mediated pathway, learning motivation significantly and negatively predicted classroom silence (*β* = −0.06, *t* = −2.20, *p* < 0.05), but the interaction term of learning motivation × teaching style did not reach statistical significance. This suggests that instructional style moderating effect on the “learning motivation → classroom silence” pathway was not statistically supported. Consequently, the mechanism through which learning motivation affects classroom silence remains relatively stable, and teaching style primarily influences classroom silence indirectly by buffering the initial damage caused by social loafing rather than by enhancing the subsequent effect of learning motivation.

1. The moderating mediating effect of teaching style, as shown in Table 8.

**Table 8:** Moderated mediation modeling tests of teaching style (*N* = 1,402).

Predictor variable	Classroom silence			Learning motivation		
	*β*	*SE*	*t*	*β*	*SE*	*t*
Constant	20.21**	1.22	16.48	−2.73*	1.26	−2.16
Social loafing	0.29**	0.02	12.99	−0.16**	0.02	−7.24
Teaching style	−0.38**	0.05	−7.07	0.79**	0.05	−15.55
Social loafing * teaching style	0.04**	0.01	7.50	−0.02**	0.01	−2.74
Learning motivation	−0.06*	0.03	−2.33			
Learning motivation * teaching style	−0.02*	0.01	−2.52			
*R* ^2^	0.27				0.26	
*ΔR* ^2^	0.26				0.25	
*F*	*F* (11,1,390) = 45.95, *p* = 0.000			*F* (9,1,392) = 54.35, *p* = 0.000		

**Denotes *p* < 0.01, *denotes *p* < 0.05.

As indicated in Table 8, Model 1 (dependent variable: classroom silence) demonstrated significant overall explanatory power (*R*^2^ = 0.27, *F* (11, 1,390) = 45.95, *p* < 0.01), and Model 2 (dependent variable: learning motivation) also exhibited significant explanatory power (*R*^2^ = 0.26, *F* (9, 1,392) = 54.35, p < 0.01), indicating good model fit to the data. Secondly, the direct effect of social loafing on classroom silence was significant (*β* = 0.29, *t* = 12.99, *p* < 0.01), suggesting that higher levels of social loafing predict increased classroom silence behaviors. Teaching style significantly moderated the relationship between social loafing and classroom silence (*β* = 0.04, *t* = 7.50, *p* < 0.01). To clarify the interaction effect, teaching style was divided into high-level (+1SD) and low-level (−1SD) groups based on ±1 standard deviation around the mean. Simple slope analysis revealed that when teaching style quality was high (+1SD), social loafing had a stronger predictive effect on classroom silence (*β* = 0.44, 95% CI [0.38, 0.50]), whereas the effect weakened when teaching style quality was low (−1SD) (*β* = 0.13, 95% CI [0.08, 0.19]).

Moderated mediation analysis further showed, in the first stage of the mediated pathway, social loafing significantly and negatively predicted learning motivation (*β* = −0.16, *t* = −7.24, *p* < 0.01), and the interaction term of social loafing × teaching style was significant (*β* = −0.02, *t* = −2.74, *p* < 0.01). This indicates that teaching style mitigated the negative impact of social loafing on learning motivation, in the second stage of the mediated pathway, learning motivation significantly and negatively predicted classroom silence (*β* = −0.06, *t* = −2.33, *p* < 0.05), and the interaction term of learning motivation × teaching style was significant (*β* = −0.02, *t* = −2.52, *p* < 0.05). This demonstrates that teaching style significantly strengthened the inhibitory effect of learning motivation on classroom silence.

Based on the Bootstrap method for testing mediating effects, the total effect of social loafing on classroom silence was 0.420 (*B* = 0.36, *p* < 0.01), of which the direct effect accounted for 88.45% (*B* = 0.32, *β* = 0.37), and the indirect effect through learning motivation was 0.04 (95% CI [0.03, 0.07]), accounting for 11.55% of the total effect. These results indicate that learning motivation partially mediates the relationship between social loafing and classroom silence. Analysis using a multi-group moderated mediation model revealed that teaching content exerted significant dual moderating effects on the social loafing-classroom silence pathway. In the first stage of the pathway (social loafing → learning motivation), the standardized regression coefficient of social loafing decreased from −0.17 to −0.15 in the high-level teaching content group (+1SD), while it remained at the initial level of −0.17 in the low-level group (−1SD). In the second stage (learning motivation → classroom silence), elevated teaching content levels strengthened the inhibitory effect of learning motivation (*β* = −0.02, *p* < 0.05). Under high-level teaching content conditions, the negative effect of learning motivation increased from −0.06 to −0.08, indicating that high-quality course content amplifies the behavioral externalization of positive learning motivation. The overall moderated mediation index was significant (Index = 0.005, 95% CI [0.001, 0.011]), demonstrating that teaching content systematically moderated the mediation model through these dual pathways. The moderating pattern of teaching methods was highly similar to that of teaching content (Index = 0.005, 95% CI [0.002, 0.012]), but its first-stage moderating effect was stronger (*β* = −0.03 vs. −0.02).

**Figure 2:**
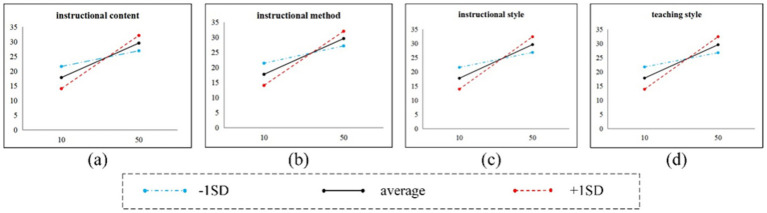
Simple slope plots.

Further simple slope plots (Figure 2) revealed that although both instructional style and teaching style belong to the pedagogical dimension, their moderating effects exhibited significant divergence. Teaching style primarily functioned in the initial impact stage of social loafing (first-stage moderation *β* = −0.03, *p* < 0.01), but did not reach significance in the behavioral transformation stage of learning motivation. Indirect effect tests showed that when teaching style was at a high level (+1SD), the mediating effect of learning motivation was 0.03 (95% CI [0.01, 0.05]), but this effect disappeared at low levels (95% CI [−0.008, 0.008]). In contrast, teacher style demonstrated comprehensive moderating capabilities: in the social loafing → learning motivation pathway, each 1-SD increase in teacher style reduced the negative effect of social loafing by 0.02 SD (*β* = −0.02, p < 0.01); simultaneously, its moderating effect on the learning motivation → classroom silence pathway was significant (*β* = −0.02, *p* < 0.05), with a moderated mediation index of 0.004 (95% CI [0.001, 0.008]). Further analysis revealed that under high-level teacher style conditions, the inhibitory effect of learning motivation on classroom silence increased by 72.3% (from *β* = −0.045 to −0.077), indicating that teacher personal characteristics play a crucial catalytic role in the externalization of motivation.

Although high-quality teaching content can suppress classroom silence through the mediating pathway (indirect effect enhancement), it paradoxically amplified the direct effect of social loafing. Under low-level teaching content conditions, the direct effect of social loafing on classroom silence was *β* = 0.14 (*p* < 0.05), whereas under high-level conditions, this effect surged to *β* = 0.43 (*p* < 0.01)—an increase of 207%. This phenomenon was also observed in the moderating models of teaching methods (low→high levels: *β* = 0.15 → 0.41) and teacher style (*β* = 0.13 → 0.44). This contradictory moderation may stem from the group differentiation effect of teaching quality: while high-quality teaching content enhances overall student participation, it may simultaneously intensify the relative deprivation experienced by students with lower learning abilities, thereby reinforcing their tendency toward silence.

## 5 Discussion

Although this study statistically tested mediation and moderation effects, the cross-sectional design precludes establishing temporal ordering. Therefore, claims regarding causal mechanisms should be tempered.

First, this study aligns with prior findings in key aspects. Our results demonstrate that social loafing significantly and positively predicts classroom silence (*β* = 0.42), consistent Frambach et al. (2014) and Zhang et al. (2019) regarding the substantial impact of students’ psychological states on classroom participation willingness. As a psychological tendency to reduce effort in group settings, social loafing diminishes students’ willingness to engage verbally. Furthermore, this study confirms the partial mediating role of learning motivation between social loafing and classroom silence, corroborating Chi and Xin (2006) and Huang et al. (2025), who likewise emphasize learning motivation as a critical psychological mechanism connecting external teaching variables with student behaviors. Additionally, instructional factors (content, methods, and style) demonstrate significant moderating effects on the “social loafing → learning motivation → classroom silence” pathway, aligning with Wendt and Courdu (2018) and Osman et al. (2022) concerning the substantial moderating role of teacher behaviors and pedagogical strategies on student engagement.

Second, this study reveals novel insights. We systematically examine the pathway mechanism of “social loafing → learning motivation → classroom silence.” While prior studies separately explored the isolated effects of social loafing (e.g., Liu et al., 2024), learning motivation (e.g., Ye et al., 2025), and classroom silence (e.g., Liu, 2020), this research innovatively integrates them into a moderated mediation model. This reveals how social loafing exacerbates silence by undermining learning motivation, enriching systematic mechanism research in this field. Crucially, our findings indicate that instructional content and methods not only buffer the first stage (social loafing → learning motivation) but also amplify motivation’s inhibitory effect in the second stage (learning motivation → classroom silence). In contrast, teaching style moderates only the first stage, while teacher style moderates the entire pathway. This differentiated moderation pattern remains unreported in prior literature, extending the single-path research by He (2005) and Guo et al. (2025) on instructional influences.

Notably, an unexpected finding emerged: While high-quality instructional content effectively enhances learning motivation’s suppression of silence, it simultaneously amplifies social loafing’s direct effect on silence. This paradoxical moderation phenomenon is scarcely documented, potentially because elevated overall teaching quality creates “high-investment–high-interaction” situational cues for all students. Yet social loafers maintain low investment, making them more likely to perceive disparities (“I contribute less than others,” “I cannot meet classroom expectations”). This horizontal comparison triggers strong relative deprivation: “I want to participate, but others respond faster and better—so I choose silence.” Such deprivation rapidly transforms into defensive silence, thereby amplifying social loafing’s direct path to classroom silence. Moreover, high-quality classroom interactions rapidly elevate “high-ability–high-engagement” students to core status positions, creating visible stratification. Teachers’ increased positive feedback toward active participants further solidifies this hierarchy, marginalizing social loafers. Consequently, higher instructional quality intensifies stratification, significantly increasing the path coefficient of social loafing’s direct effect on silence.

Beyond social loafing and instructional factors, Confucian cultural norms provide profound legitimization for silence, partially explaining why this study observed an amplified direct effect of social loafing on silence (*β* increased from 0.14 to 0.43) under high-quality teaching conditions. As cited in the literature Lv (2020), Shi (2020), traditional norms emphasizing prudent speech, teacher authority, and collective harmony construct silence as a virtue for respecting authority and avoiding conflict. In high-interaction, high-expectation classrooms, public expression carries elevated value. Consequently, superior teaching quality intensifies social comparison, activating Confucian silence norms as rationalizing mechanisms for social loafing. This aligns with Frambach et al. ‘s (2014) cross-cultural finding that students in teacher-reverent Confucian classrooms perceive silence as an active choice rather than passive deficiency.

Additionally, confucian traditions not only confer moral legitimacy to silence but also reinforce students’ self-positioning as inferiors through high power distance and hierarchical structures. As noted by Dong and Zhao (2023), traditional Chinese classrooms perpetuate a “teacher-dominated discourse system” that socializes students into being “listeners” rather than “questioners.” Even in modern universities, teacher-initiated Q&A sessions remain implicitly framed as “superiors granting performance opportunities to inferiors” (Zhang and Shi, 2020). Within this hierarchy, the frequent interaction opportunities afforded by high-quality teaching may be interpreted by loafing students as “scrutiny from superiors” rather than “egalitarian dialogue,” fostering the psychology: “The more excellent the teacher, the more I must refrain from speaking freely to preserve hierarchical order.” This mechanism parallels Muuro et al. ‘s (2014) finding that “power perception inhibits online collaboration”—when students perceive teachers as having absolute evaluative power, even technologically enabled platforms fail to motivate low-self-efficacy individuals to speak. Our results further indicate that power distance does not automatically diminish with improved teaching techniques. Paradoxically, high-quality classrooms magnify performance disparities and evaluation visibility, intensifying status threat among loafing students in high-power-distance cultures. Consequently, they resort to silence to maintain psychological safety within the hierarchical structure.

## 6 Conclusions and recommendations

### 6.1 Conclusion

1. Learning motivation plays a partial mediating role in the relationship between social loafing and college students’ classroom silence behavior. Social loafing not only directly and positively predicts classroom silence but also indirectly exacerbates such behavior by undermining learning motivation. That is, diminished learning motivation constitutes a key psychological mechanism through which social loafing translates into classroom silence, though other potential mediating factors may also exist.
2. All four dimensions of pedagogical factors significantly moderate the pathway from social loafing to classroom silence, yet their moderating patterns differ. Teaching content and teaching methods moderate the mediation model through dual pathways: they simultaneously mitigate the negative impact of social loafing on learning motivation and strengthen the inhibitory effect of learning motivation on silence. Teaching style functions only in the first-stage moderation (social loafing → learning motivation), while teacher style systematically moderates both stages (social loafing → learning motivation and learning motivation → classroom silence) of the mediation pathway.
3. Although high-quality pedagogical factors suppress classroom silence through the mediating pathway, they paradoxically amplify the direct effect of social loafing. That is, improvements in teaching quality may be accompanied by a group differentiation effect: while enhancing classroom engagement for most students, high-level teaching may simultaneously intensify the silence tendency among individuals with pronounced social loafing tendencies.

### 6.2 Recommendations

1. Incorporate a “student-led practice module” into the existing curriculum system, requiring each course to include at least 1–2 small-scale tasks designed by students (e.g., 10-min micro-lesson presentations, case study research proposals). Additionally, integrate classroom interaction data into regular assessment weighting and establish a dynamic optimization mechanism for pedagogical elements. Collect anonymous mid-semester feedback to promptly adjust teaching strategies according to student needs.
2. Implement teacher development training programs to enhance educators’ sensitivity in identifying signs of social loafing and foster inclusive classroom atmospheres. Mandate a “rotating role system” for group assignments by defining fixed roles (e.g., data collection, PPT creation, presentation delivery) and utilizing randomized allocation tools to ensure each student undertakes at least two distinct roles.
3. Discipline-specific pedagogical optimization: For theoretically intensive courses, prioritize enhancing the relevance and practicality of teaching content to counteract motivation erosion caused by social loafing through heightened content value. Practice-oriented courses should emphasize innovative teaching methods (e.g., project-based learning, flipped classrooms), leveraging high-interactivity designs to activate student engagement.

### 6.3 Research limitations and future directions

1. Cross-sectional design constraints: Despite testing path significance through structural equation modeling, the single-wave questionnaire design precludes establishing temporal precedence or causal direction between variables. In the future research, we can implement longitudinal designs (e.g., cross-lagged panel models) or pre-post interventions with measurements at Weeks 4, 8, and 16 to track dynamic trajectories of social loafing and silence.
2. Nesting effects unaddressed: Students within the same class share instructional contexts, yet individual-level analysis ignored classroom-level clustering. This may underestimate standard errors due to within-class homogeneity. In the future research, we can employ hierarchical linear modeling nesting students within classes/teachers to partition individual- and classroom-level variance, enabling examination of cross-level moderation (e.g., teaching style effects on social loafing → silence slopes).

## Data Availability

The data analyzed in this study is subject to the following licenses/restrictions: the data that support the findings of this study are available from the corresponding author upon reasonable request. Requests to access these datasets should be directed to shanshanli0809@163.com.
